# Risk of Permanent Dysfunction of Facial Nerves After Open Rigid Internal Fixation in the Treatment of Mandibular Condylar Process Fracture

**DOI:** 10.3390/medsci13030121

**Published:** 2025-08-09

**Authors:** Paulina Agier, Marcin Kozakiewicz, Szymon Tyszkiewicz, Izabela Gabryelczak

**Affiliations:** 1 Multispeciality Dental Clinic, 106/116 Kośćiuszki Av., 90-442 Łódź, Poland; 2 Department of Maxillofacial Surgery, Medical University of Lodz, 113 Żeromskiego St., 90-549 Łódź, Poland; marcin.kozakiewicz@umed.lodz.pl (M.K.); szymon.tyszkiewicz@umed.lodz.pl (S.T.)

**Keywords:** condyle, mandible, fracture, open treatment, surgery, open rigid internal fixation, osteosynthesis, complication, facial nerve, rehabilitation

## Abstract

Background: Facial nerve palsy is a relatively common complication following open rigid internal fixation (ORIF) of a mandibular condylar fracture. The aim of this study was to investigate the risk factors that influence post-operative facial nerve function and the recovery process. Methods: A retrospective study was conducted based on the medical records of 329 patients who underwent ORIF treatment for condyle fractures, with the follow-up period being 24 months long. Results: During the initial post-operative examination, 50.45% of patients exhibited some signs of facial nerve dysfunction, ranging from slight to severe, and 48.63% of patients presented transient palsy, while only 1.82% presented permanent facial nerve palsy. Female patients were found to be more susceptible to post-operative facial nerve palsy. Patients with multiple mandibular fractures and bilateral condyle fractures had a worse prognosis. The preauricular approach and its modifications were identified as posing the greatest risk to the facial nerve. The safest approach was the retromandibular approach. Patients treated for injuries resulting from traffic accidents or falls had a worse prognosis than those treated for assault injuries. Conclusions: Post-operative facial nerve palsy following ORIF of the mandibular condyle is, in most cases, transient and can be effectively treated. However, it is important to choose the safest possible surgical approach. The safer approach, the retromandibular approach, should be considered when possible.

## 1. Introduction

For the treatment of mandibular condylar process fractures, the doctor theoretically has two options: closed treatment or open treatment. But practically, there are specific considerations for the choice of treatment method [1]. This is not a randomised approach. In evaluating the efficacy of open reduction and internal fixation (ORIF) and closed conservative treatment, the primary consideration is the comparative analysis of the advantages and disadvantages inherent to each method. The adverse effects of the treatment selected can have a significant impact on patients’ quality of life.

There are some advantages to conservative treatment of mandibular condylar process fractures, such as avoidance of post-operative infection, haematoma, or visible scarring on the facial skin. However, these are of lesser clinical importance [2,3,4]. Evaluating the long-term outcomes of closed treatment, it has been found that it often does not give good results. It fails in the treatment of fractures with significant displacement, with full dislocation, and especially in old fractures. Improper reduction of bone fragments (or no reduction) causes a series of disorders of the masticatory organ [5]. Many studies have shown that open surgical treatment has better results than closed treatment. Patients who have undergone surgery experience less of a decrease in vertical ramus height than patients who have undergone closed treatment. Additionally, the results of post-operative mobility of the temporomandibular joint and accuracy of the occlusion and entire function are significantly better in patients treated surgically than in those treated conservatively [1,2,3].

Nowadays, the scope of indications for conservative treatment is limited. This method can be indicated for paediatric or significantly compromised patients when general anaesthesia is contraindicated. It can also be applied in cases when occlusion is slightly changed and in fractures without significant displacement, i.e., where there is direct contact between injured bone fragments [6,7]. The widely accepted eligibility criteria for open treatment are as follows: proximal fragment deviation more than 30^0^, mandibular ramus shortening more than 2 mm, significant malocclusion, intracapsular haematoma in the temporomandibular joint (TMJ), and laceration of the external acoustic meatus [8,9]. Most cases of displaced fractures seen today are treated via ORIF with the application of one of more than 50 types of plates and dozens of types of screws [10,11,12,13,14,15,16]. Thus, open treatment facilitates the restoration of anatomical skeletal relationships, but the biological price for this advantage is the surgical risk associated largely, in this case, with the function of the facial nerve. Nerve palsy was described directly after osteosynthesis in 10% [17], 17% [3,18], 20% [2], or 48% [19] of patients in previous studies. This seems to be an underestimated number, and a crucial one. It is worth mentioning that the fracture of the mandibular condyle head, which is a direct element of the TMJ, is the most difficult type of mandibular process fracture to fix. The incidence of transient facial nerve palsy observed immediately after TMJ surgery is notably high, with reported rates ranging from approximately 71% [20] to even 100% in some series [21]. The facial nerve is divided into five branches: the temporal, zygomatic, buccal, marginal mandibular, and cervical branches. According to the literature, when treating condylar fractures, the temporal and zygomatic branches are at the highest risk of iatrogenic injury and post-operative facial nerve dysfunction. The risk of injury to the buccal branch is moderately high, particularly during retromandibular and transmasseteric approaches [22,23,24]. Facial nerve palsy causes considerable facial disfigurement and emotional distress to the patient. It can cause detrimental effects on both voluntary and involuntary actions of facial muscles, including the closing of the eyelids and drying out of the cornea. Importantly, it can interrupt normal daily functions such as eating and drinking, too. Facial nerve palsy is a serious condition, but today it can be treated effectively in many ways, from early post-operative care to surgical management [25,26,27]. However, a review of the literature suggests that facial nerve weakness as a post-operative complication is mostly temporary [3].

The aim of this study was to examine potential risk factors impacting post-operative facial nerve function and the recovery process. The authors would like to explore how to reduce the risk of post-operative facial nerve dysfunction and how to deal with post-operative facial nerve palsy.

## 2. Materials and Methods

Retrospective observational data were collected from the medical records of patients at the Department of Maxillofacial Surgery at the Medical University of Lodz in Poland. Reporting of the study followed the STROBE checklist [28].

**Figure 1:**
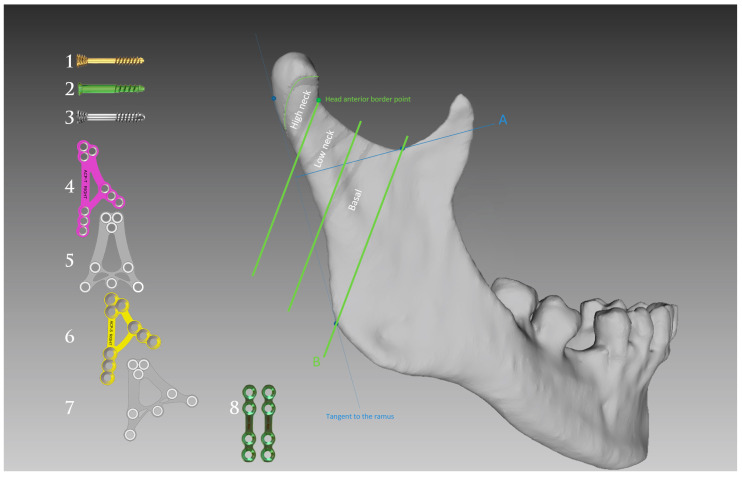
Applied fixation materials for open rigid internal fixation of mandibular condyle. Mandibular head fragments were put together using a titanium compression headless screw (**1**), a titanium lag screw (**2**), or a magnesium compression headless screw (**3**). The application of the material below the mandible head depended on the level of the condylar process fracture and the width of the bone surface (it was a titanium assortment). High-neck fractures were osteosynthesised with an ACP-Tall plate (**4**) or angle-inserted screws (**1**–**3**). Low-neck fractures were fixated with XCP universal plates (**5**) or ACP-Short plates (**6**). Basal fractures were fixated with patient-side dedicated XCP (**7**) or ACP-Short (**6**) plates. When it was not possible to fit a dedicated plate to the morphology of the fracture and the course of the fracture line, straight plates were used (**8**). Fractures were divided into head fractures, high-neck fractures, low-neck fractures, and basal fractures according to the presented oblique classification lines [29]. A - the sigmoid notch line (Loukota’s line); B - the line along the most prominent point of the posterior border of the masseteric tuberosity and the deepest point of the sigmoid notch.

Clinical material was collected from 2017 to 2023 based on the following inclusion criteria: maxillofacial injury, mandible condyle fracture, surgical treatment, and complete medical documentation. The exclusion criteria were used: close treatment (i.e., conservative treatment), pre-operative facial nerve palsy, failure to report for follow-up examinations, or treatment refusal. Overall, our sample comprised 329 patients aged 40 ± 16 years old (median 37 y.o.). All these patients were treated surgically (Figure 1) in an average time after injury of 9 ± 12 days (median 7 days).

A comprehensive data set was collected, including details such as the patient’s sex, age, place of residence, incidence of patients referred from other medical centre, number of comorbidities, white blood cell count, peripheral venous blood haemoglobin level, use of stimulants, waiting time from injury to surgery, reason for injury, the type of mandibular condylar process fracture, duration of the surgical procedure, number of treated mandibular fractures during the surgical procedure, the incidence of intraoperative interruption of facial nerve branch, the surgical approach, and the type of fixing material used.

The categorization of mandibular condylar process fractures was conducted in accordance with Kozakiewicz’s classification [29] and Neff’s nomenclature [30,31], meaning fractures were categorised into six distinct diagnoses: base, low-neck, high-neck, head type A, head type B, and head type C.

The surgical procedure was preceded by the administration of pre-operative antibiotic prophylaxis, which most commonly comprises amikacin, metronidazole, and ampicillin (due to frequent presentation of additional open fracture in mandible body). The surgical procedure was performed under general anaesthesia, with nasal intubation, submental intubation, or tracheostomy intubation (depending on additional injuries). No intramaxillary fixation (IMF) was used during fixation of the mandibular condyle. The surgical approach selected for the patient was contingent upon the fracture location. Eight accesses were utilised: extended temporally preauricular, retroauricular, preauricular, auricular, retromandibular, extended preauricularly retromandibular, periangular, and intraoral. Nerve monitoring was not used during surgery. As long as it was possible, the facial nerve branches were not intentionally dissected when operating on basal or low-neck fractures (to avoid extensive stretching). However, in most cases of high-neck and mandibular head fractures, the facial nerve branches were encountered, and there was a need to dissect them on the length of 1–3 cm to avoid iatrogenic nerve injury during the fracture fixation. This dissection was performed according to the anatomical location of the facial nerve and by following the Yang and Yoo classification [32]. In the event of an interruption to the continuity of a nerve branch, both nerve ends were connected immediately. In such cases, the interrupted branch was connected by sutures, end to end, without stretching and with microscope assistance.

The bone fixation material selected was appropriate to the fracture type (Figure 1), with various materials employed in different cases: compressive screws, XCP plates, ACP plates, and straight plates (ChM, Juchnowiec Kościelny, Poland, www.chm.eu, access on 1 August 2025). Intravenous 16–24 mg dexamethasone was administered for 2–3 days.

**Figure 2:**
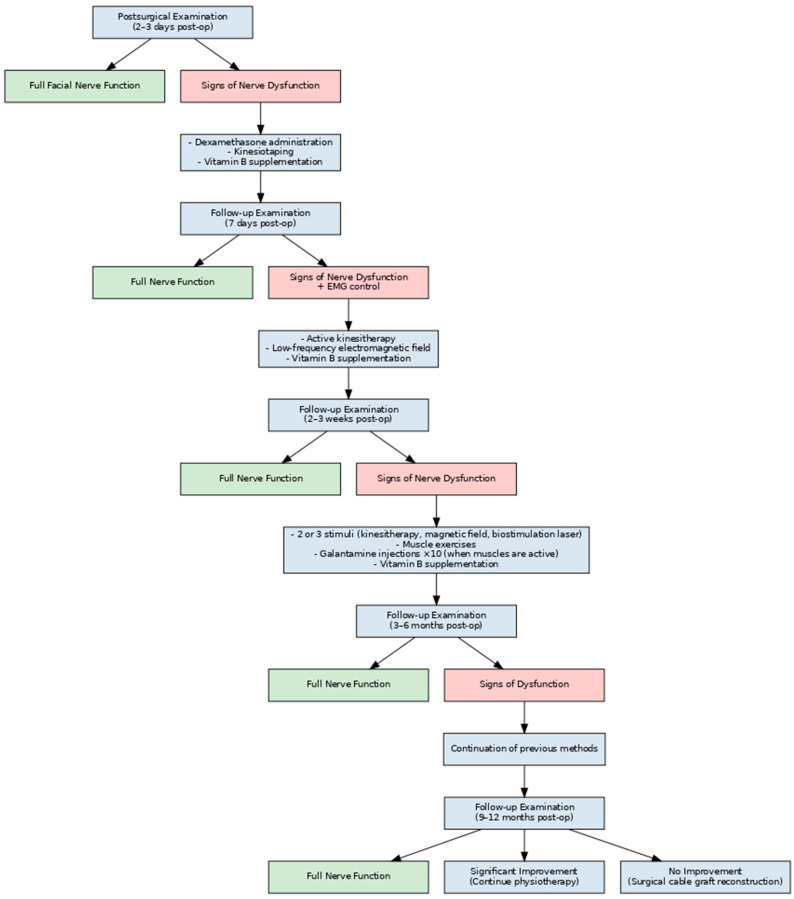
Flowchart showing an applied protocol for the treatment of post-operative facial nerve dysfunction.

Therapeutic management after surgery regarding facial nerve function included provision for deficits that occurred in the patient (Figure 2). The evaluation of the extent and pattern of possible disorders was performed on three occasions, i.e., on day 0, on day 2–3 post-operatively (i.e., when the full volume of post-operative swelling appeared), and on day 7, when the full clinical manifestation of facial nerve weakness was visible. It was evaluated whether there was mechanical damage to the branches of the nerve or only weakness of function itself. The clinical evaluation of facial nerve dysfunction was classified according to the House–Brackmann scale.

**Figure 3:**
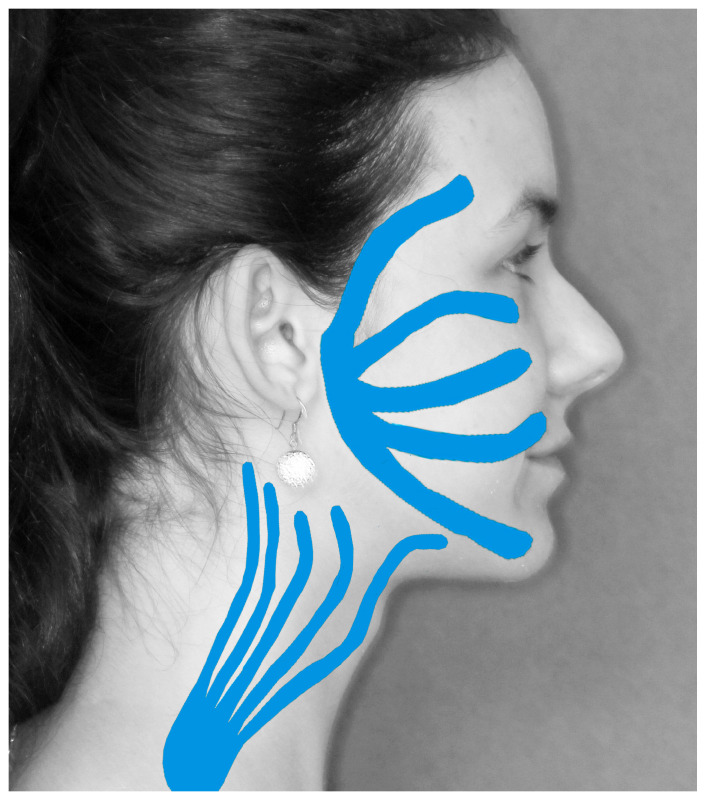
Method of Kinesiotaping application after open reduction and rigid fixation of mandible condyle.

The initial element of improvement applied to patients was anti-oedema therapy with dexamethasone. Kinesiotaping (Figure 3) was applied. From the first day that facial nerve dysfunction was identified, supplementation with vitamins B1, B6, and B12 was administered.

From the seventh day, when the characterised cause of dysfunction and its extensiveness were known, stimulation adapted to the process of regeneration (when the axon was not damaged) or reinnervation (when the injury concerns the reconstruction of the course of the axon) of the fibres of the affected nerve was started. If there was no unrepaired neurotmesis in the above evaluation, active kinesitherapy (movement improvement) and low-frequency electromagnetic field magnetotherapy treatments were started. Exercise was the next stage of improvement. The facial muscles were actively exercised, respecting the principles of physiology. When some facial muscle activity was already present, 5 mg galantamine, as an acetylcholinesterase inhibitor, was administered subcutaneously in six 10-injection series repeated every 2 weeks (1 administration of the drug every 1 day).

Physiotherapy was continued until results were found to be satisfactory or until complete recovery of nerve function. If the function of the nerve had not recovered after 6 months, another attempt at verification was made to determine whether nerve damage had occurred. The reason for the lack of improvement in nerve function was determined. If there was no interruption, stimulation of the nerve with no more than 3 stimuli (kinesitherapy, magnetic field application, or laser biostimulation) was continued. If it was assessed that neurotmesis had occurred, a cabled nerve graft would be considered (as the House–Brackmann scale was 4–6).

Facial nerve function was measured according to the House–Brackmann scale during post-operative hospitalization and at 1, 2, 3, 4, 5, 6, and 24 months after surgery. The House–Brackmann scale has six levels of nerve dysfunction (Table 1). The scores range from 1 (full nerve function) to 6 (total loss of nerve function). The same assessment and treatment protocol was employed for all patients. Modifications to physiotherapeutic techniques could be made in some cases due to patients’ comorbidities and other health conditions.

Statistical analysis was performed in Statgraphics Centurion 18 (Statgraphics Technologies Inc., The Plains City, Warrenton, VA, USA; www.statgraphics.com, accessed on 1 August 2025). Statistical analysis consisted of a normality check, a paired-sample sign test for evaluation of time-dependent alterations, the Kruskal–Wallis test for evaluation of the influence of qualitative factors on facial nerve function, and a linear regression analysis performed for assessment of quantitative variable relationships. A *p*-value less than 0.05 was considered statistically significant.

## 3. Results

**Figure 4:**
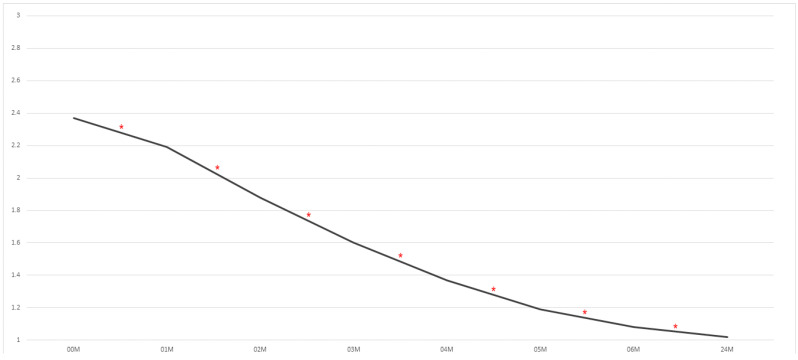
A summary of the results of our evaluation of peripheral facial nerve function impairment after open reduction and rigid fixation involving the condylar process of the mandible. The vertical axis shows the House–Brackmann scale (with extremes of 6—total loss of facial nerve function—and 1— full function). The horizontal axis shows the time intervals at which the patients were examined. A statistically significant improvement (*p* < 0.001) was noticed between all study timepoints. Abbreviations: 00M—immediately post-op; 01M, 02M, 03M, 04M, 05M, 06M, and 24M—1, 2, 3, 4, 5, 6, and 24 months post-op, * - an asterisk indicates a statistically significant difference between neighborly average values.

The applied protocol effectively eliminates facial nerve dysfunction within 4–6 months after the completion of surgical treatment. During the follow-up period, complete facial nerve function was restored in 98.16% of patients with this complication (Figure 4). The remaining two patients had a weakness grade of 3 on the House–Brackmann scale, and four had a weakness grade of 2 at 24 months after the operation. In this study, at 6 months after surgery, 94% of patients experienced full restoration of function. The percentages for the other timepoints are as follows: after 5 months: 84%; after 4 months: 76%; after 3 months; 63%; after 2 months: 53%; after 1 month of rehabilitation: 38%. Full facial nerve function in the first few days after surgery was found in 29% of patients.

The included cohort was based on 329 medical records. The initial post-operative examination revealed signs of facial nerve palsy in 50.45% of patients. In 48.63% of cases, the palsy was transient; only 1.82% were classified as having permanent facial nerve palsy. It is important to analyse these six cases of incomplete recovery. They were individuals between the ages of 41 and 59. We had four males with type C fractures of the mandibular head (two of them suffered from high blood pressure and were successfully treated for it) and two females with base fractures. Four of these patients were completely healthy in terms of internal medicine. They were operated on between 5 and 10 days after injury. The surgical approach used involved preauricular retromandibular extension (basal fracture) or a preauricular temporal extended by hockey stick line (mandibular head fracture). No damage to the facial nerve branch was found intraoperatively. All of these patients were able to close their eyes from the third month after surgery (grade 2 or 3 on the House–Brackmann scale, with such scores remaining until the end of the follow-up period in this study); thus, there was no risk of drying out the cornea. It is worth noting that some (not much) progress of facial nerve recovery in many patients still occurs 6 months after surgery, and further functional improvement is noticeable 2 years after operation. All obtained data are presented in Table 2.

**Figure 5:**
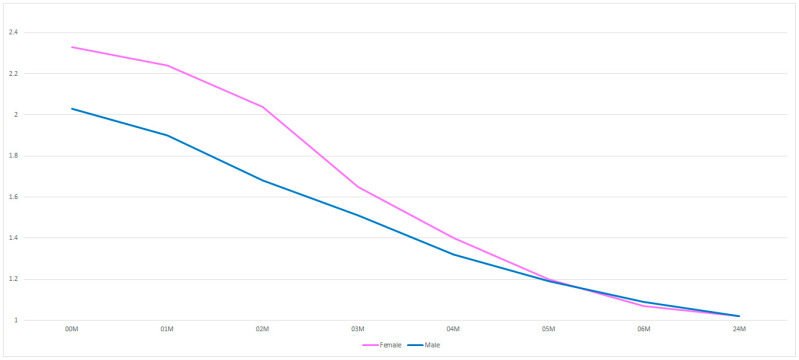
Sex-dependent reduction in facial nerve weakness. The vertical axis shows the House–Brackmann dysfunction scale (with extremes of 6—total loss of facial nerve function—and 1— full function). The horizontal axis shows the time intervals at which the patients were examined (in months).

It appears that the facial nerve in female patients is more sensitive to stimuli triggered during surgery of mandibular condylar process fracture (Figure 5). This is evident up to 4 months of post-operative rehabilitation. Thereafter, functional status improves equally in males and females.

**Figure 6:**
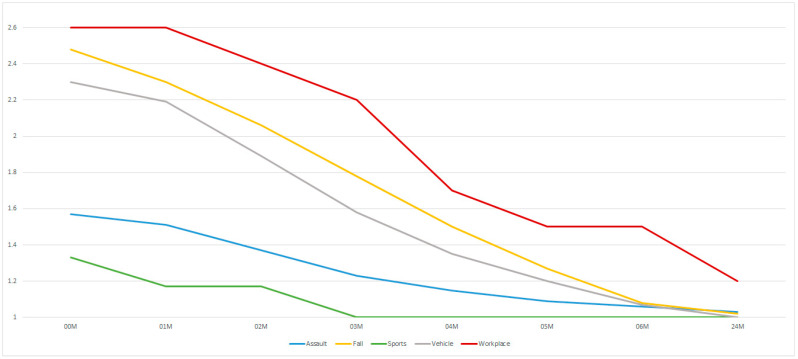
Reason for injury. The vertical axis shows the House–Brackmann scale (with extremes of 6—total loss of facial nerve function—and 1—full function). The horizontal axis shows the time intervals at which the patients were examined (in months).

Greater dysfunction of the VII cranial nerve occurs (*p* < 0.05) in treating patients after traffic accidents and falls versus treating the effects of assaults (Figure 6). These differences begin to fade at 4 months after surgery, when function improves in patients after vehicle accidents. Subsequently, any differences disappear.

**Figure 7:**
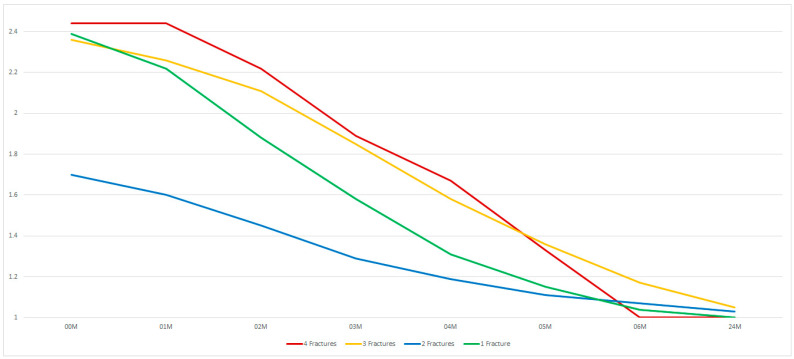
The effect of the number of treated fractures on facial nerve function impairment. The vertical axis shows the House–Brackmann scale (with extremes of 6—total loss of facial nerve function—and 1—full function). The horizontal axis shows the time intervals at which the patients were examined (in months).

An increased number of fractures in the mandible worsens the condition of the facial nerve after osteosynthesis of all fractures (*p* < 0.05). It was noted that the best early results (up to 4 months after surgery) were obtained with double fractures rather than single fractures (Figure 7).

Bilateral condyle fractures have a worse prognosis and significantly slower resolution of facial nerve dysfunction (*p* < 0.05). It can be seen up to 6 months after surgery, and the impact of this factor disappears as late as at 24 months of follow-up.

**Figure 8:**
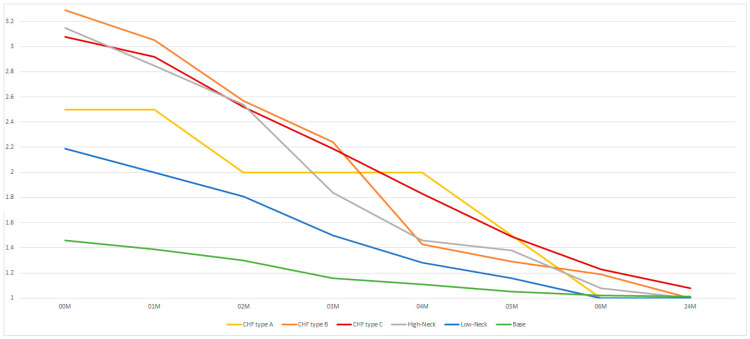
The influence of treatment of a specific type of fracture on post-operative facial nerve function. The vertical axis shows the House–Brackmann scale (with extremes of 6—total loss of facial nerve function—and 1—full function). The horizontal axis shows the time intervals at which the patients were examined (in months).

Treatment of mandibular head fractures (B and C) and high-neck fractures (Table 3) result in greater stress on the facial nerve during osteosynthesis (*p* < 0.05). From the 4th month after the operation, the slower rate of return of facial muscle function is seen only after treatment of type C mandibular head fractures. The final effect (the same in all the diagnoses studied) is seen in the sixth post-operative month. This effect persists at the twenty-four-month follow-up timepoint (Figure 8).

**Figure 9:**
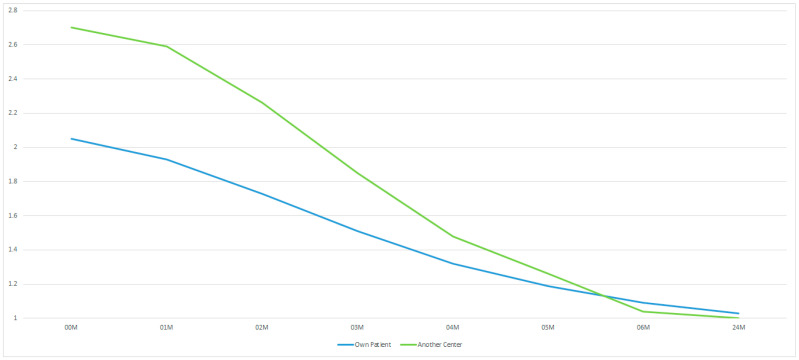
The results for our patients and those arriving from elsewhere in the country. The vertical axis shows the House–Brackmann scale (with extremes of 6—total loss of facial nerve function—and 1—full function). The horizontal axis shows the time intervals at which the patients were examined (in months).

Admission to the department and treatment of a patient from another medical centre is associated with longer recovery in the area of facial muscles (*p* < 0.05). This difference disappears permanently from the 4th month after the surgery (Figure 9).

**Figure 10:**
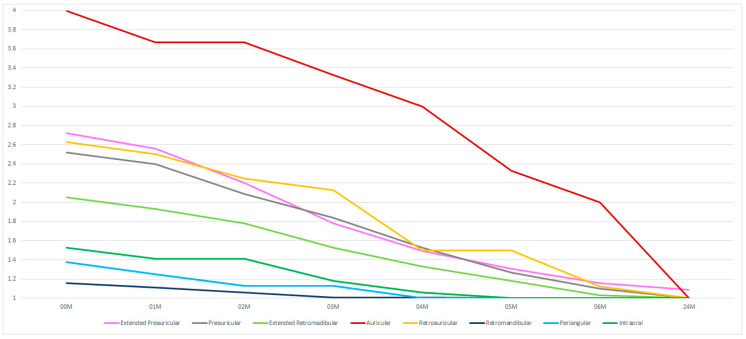
The surgical approaches used. The vertical axis shows the House–Brackmann scale (with extremes of 6—total loss of facial nerve function—and 1—full function). The horizontal axis shows the time intervals at which the patients were examined (in months).

Preauricular approaches (including extended and auricular approaches) are associated with greater effects on the action of the facial nerve (*p* < 0.05) (Table 4). However, within 24 months, it is possible to achieve the same result of 1 on the House–Brackmann scale in patients treated with all approaches (Figure 10).

**Figure 11:**
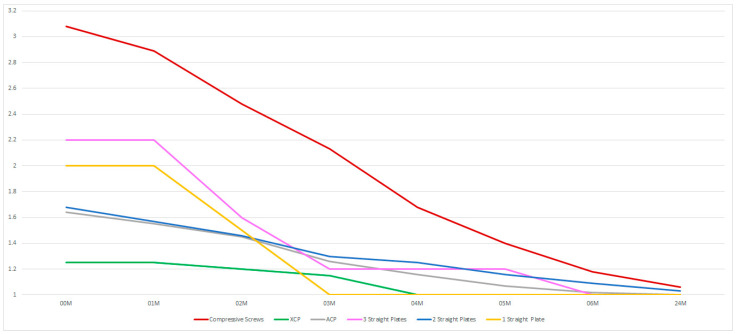
The fixing materials used. The vertical axis shows the House–Brackmann scale (with extremes of 6—total loss of facial nerve function—and 1—full function). The horizontal axis shows the time intervals at which the patients were examined (in months).

The cases wherein the compression screws were used were related with greater impairment of facial nerve function (*p* < 0.05), which requires rehabilitation longer than 6 months (Figure 11).

When assessing intraoperative interruption of the nerve branch, one can immediately notice clearly greater dysfunction of the nerve in comparison to the cases without neurotmesis (*p* < 0.05). After 1 month, the effects of treatment were equalised and, finally, all patients affected by neurotmesis emerged from the treatment without any complications.

**Figure 12:**
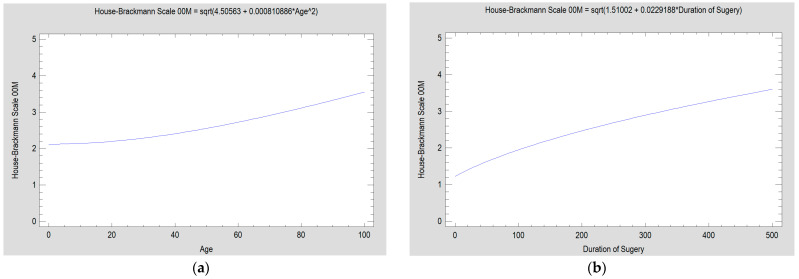
Factors having a negative impact (*p* < 0.05) on the function of the facial nerve after surgery: (**a**) patients’ age (in years); (**b**) duration of surgical procedure (in minutes).

**Figure 13:**
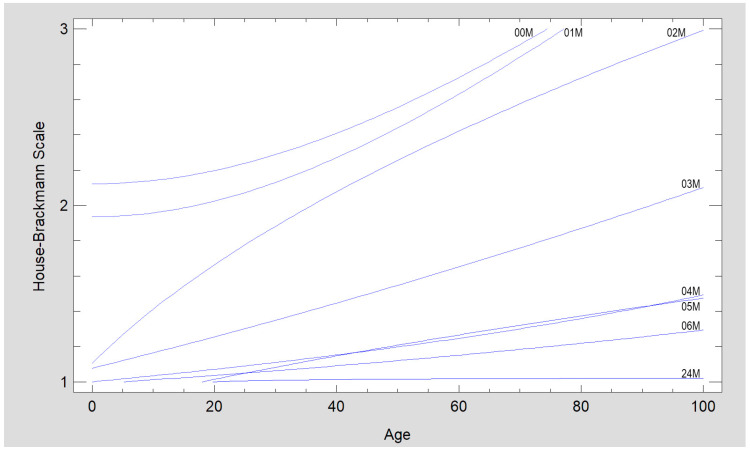
The age dependence of facial nerve impairment disappears over time (it is a relatively weak relationship). The fastest improvement occurs up to 4 months after surgery. Regression equations and correlation coefficients are given below: House–Brackmann scale 00M = sqrt(4.50563 + 0.000810886 × Age^2^), *p* < 0.001, CC = 0.20; House–Brackmann scale 01M = sqrt(3.7447 + 0.000882675 ×Age^2^), *p* < 0.001, CC = 0.23; House–Brackmann scale 02M = sqrt(1.21885 + 0.0773657 × Age), *p* < 0.001, CC = 0.25; House–Brackmann scale 03M = (1.03791 + 0.0041188 × Age)^2^, *p* < 0.001, CC = 0.21; House–Brackmann scale 04M = 1/(0.999944 − 0.00329891 × Age), *p* < 0.001, CC = 0.22; House–Brackmann scale 05M = 1/(1.01257 − 0.002396 × Age), *p* < 0.001, CC = 0.20; House–Brackmann scale 06M = sqrt(0.742555 + 0.0143199 × Age, *p* < 0.01, CC = 0.15; House–Brackmann scale 24M = 1/(0.974871 + 0.496922/Age), *p* = 0.124, CC = 0.09; Abbreviations: 00M—immediately post-op; 01M, 02M, 03M, 04M, 05M, 06M, and 24M—1, 2, 3, 4, 5, 6, and 24 months post-op; CC—correlation coefficient.

The patient’s place of residence, use of stimulants, and number of comorbidities do not affect rehabilitation progress. Waiting time for treatment did not affect the resulting facial expressions in these studies, but this is because the authors aim to operate on patients within 1–2 weeks after injury. Similarly, white blood cell count has no effect on facial nerve function. In contrast, the condition of the facial nerve immediately after treatment was affected by the age of the patients and the length of the surgical procedure (*p* < 0.05) (Figure 12 and Figure 13).

The severity of facial nerve dysfunction is in direct proportion to the patient’s age. Older patients exhibit greater dysfunction. This weak relationship occurs during early rehabilitation (00M–05M, *p* < 0.001; 06M, *p* < 0.01) and disappears in the distant period (24M, *p* = 0.124), indicating that, regardless of age, every surgically treated patient can expect facial nerve dysfunction to resolve (Figure 13).

**Figure 14:**
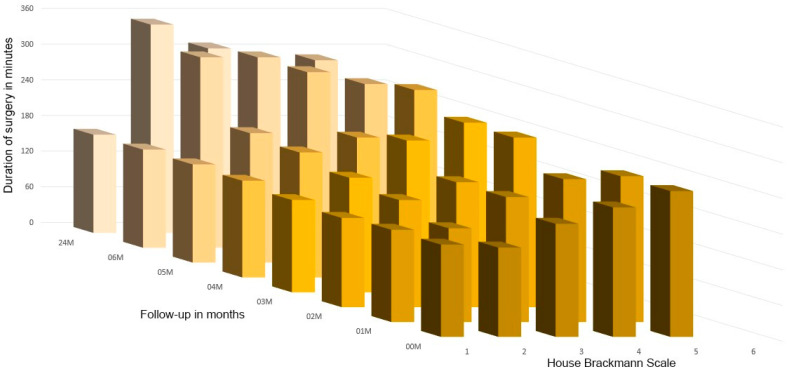
The impact of the duration of surgery (in minutes) on post-operative facial nerve function, measured in accordance with the House–Brackmann scale during follow-up (in months). Facial nerve dysfunction is strongly related to the duration of the surgical procedure. Longer procedures induce greater dysfunction (*p* < 0.001 for study timepoints up to 6 months after surgery). This relationship weakens slightly (*p* < 0.01) at 24 months after operation as a result of successful rehabilitation (only single patients with dysfunction of grade 2 and 3 remained; the rest recovered completely). Abbreviations: 00M—immediately post-op; 01M, 02M, 03M, 04M, 05M, 06M, and 24M—1, 2, 3, 4, 5, 6, and 24 months post-op.

It has been observed that an increase in the duration of surgery is associated with an elevated probability of post-operative facial nerve dysfunction (00M *p* < 0.001) (Figure 12b and Figure 14). The research also indicated a relation between the duration of surgery and the recovery of the facial nerve during follow-up. This relation was found to be significant in the first month and in the period of treatment after the third month post-op (01M, *p* < 0.001; 04M, *p* < 0.001; 05M, *p* < 0.001; 06M, *p* < 0.001; 24M, *p* < 0.01). It was observed that patients who underwent surgery for a longer duration demonstrated a worse functional capacity of the facial nerve (Figure 14).

Moreover, the better the haematological parameters before surgery (venous blood haemoglobin level), the less facial nerve dysfunction after surgery (*p* < 0.05).

## 4. Discussion

It can be adequately demonstrated that the occurrence of dysfunction of the facial nerve depends on the experience of the operator, the location of the fracture (high-neck fractures are dangerous), dislocation of the proximal fragment [18], the choice of surgical approach (traditional submandibular and retroparotid jeopardise the nerve), and the patient’s gender (female patients have a worse prediction) [33]. From this study, the authors can further add that falls and workplace injuries appear to provide the most difficult fractures, the treatment of which temporarily but significantly weakens the function of the facial nerve. Bilateral condylar fractures cause longer neurological recovery too. This is probably due to the overall greater trauma.

It is important to remember that osteosynthesis in condylar process fractures is not just surgery of the base of the condylar process, although it is undoubtedly the most common fracture in this region [34]. A significant group are fractures of the mandibular head. This is important for functional reasons, as there is a risk of ankylosis, osteoartrosis, and cranio-mandibular dysfunction [1]. And at this height of the condyle, temporomandibular joint surgery is required. According to the literature, in these procedures, the risk of transient facial nerve dysfunction increases up to 71–100% [20,21].

It should be noted that, in terms of treatment burden, high-neck fractures are more similar to fractures of the mandibular head than to fractures of the lower part of the condylar process. This is undoubtedly due to the technical difficulties posed by the fixation of high-neck fractures of the mandible [35]. It is the narrowest part of the condylar process, where it is often difficult to fit a plate, and it is difficult to find a place for 3–4 screws to sit in the proximal bone fragment [36]. Often, osteosynthesis methods developed for the mandibular head are used here [37,38], or long screws (Figure 1: material 2 and 4) guided through the upper holes of the plate are combined, and those screws are anchored in the mandibular head [5,39]. The difficulty of reduction and fixation in this fracture generates a significant percentage of initial facial nerve weakness.

The choice of approach is based on the type and location of the condylar fracture. It is essential to choose the proper surgical approach for the type of fracture. An unsuitable approach can increase the risk of facial nerve injury. In the case of basal fractures, many surgical approaches have been applied for the procedure. These include the retromandibular approach (with its different variations, such as the transparotid, retroparotid, or preparotid approaches), the periangular approach, the upward extended retromandibular approach, and the intraoral approach (with endoscope assistance). The authors strongly encourage limiting the choice to the intraoral or the retromandibular upward extended approaches. In the case of fractures of the condylar neck, the preauricular approach is applied. In many cases of treatment for low-neck fractures, the preauricular approach extended downwards is recommended. High-neck fractures indicate the utilization of the preauricular approach, extended upwards to protect facial nerve branches. Surgeons specializing in the treatment of fractures of the condylar head are faced with the challenge of selecting the most appropriate approach for their surgical intervention. These approaches can be categorised as auricular, preauricular, or retroauricular [9,40,41,42]. Our study revealed that, of all the approaches examined during the investigation, the temporally extended preauricural or the retrauricular approaches were considered relatively safe for the facial nerve. In addition to deciding on the appropriate surgical approach, it is crucial to select the proper method of intraoperative facial nerve protection. According to Al-Moraissi et al., nerves that are dissected during surgery are more stretched and have a poorer blood supply than undissected nerves. This can lead to disturbed post-operative nerve function [22].

Analysing the relationship between surgical approach and the state of the facial nerve after the procedure, two groups of approaches can be observed: 1. minimally traumatic (the retromandibular, the periangular, and the intraoral are selected most often for fixation of the base of the mandibular condyle and low-neck fracture) and 2. more traumatic (preauricular, retroauricular, and preauricular extended) [43]. Somewhere between them is the retromandibular extended approach, which should not be surprising because it is characterised by the extension of the minimally traumatic retromandibular approach towards the more traumatic preauricular approach [22,44]. The superficial surgical approaches (i.e., transparotid, transmasseteric anteroparotid, and high perimandibular approaches) are known to be safe in treatment, especially for female patients with dislocated condyles [33], probably due to lower traction of soft tissue. Moreover, Al-Moraissi et al. demonstrated that the anterioparotid approach is safer than the transparotid approach [22]. It is worth noting that, in the present study, it was found that the intraoral approach causes greater weakening of the facial nerve (although slight) than the retromandibular approach. The greater trauma observed with the auricular approach is a surprise, though this approach is a modification of the preauricular approach. Neurological rehabilitation requires maintenance for more than 6 months. It seems that this may be due to the significant distal tissue entry, causing 1 cm more exposure of the facial nerve branches than in the preauricular approach. Our study indicated that the periauricular approach is associated with an increased risk of transient post-operative facial nerve palsy. The literature also notes that, when using the periauricular approach, the facial nerve remains at risk of injury [41,42]. On the other hand, the interruption of the nerve branch itself does not seem to be significant for function if it is identified and repaired. Nerve branch anastomosis is an effective method of treating an accidentally severed branch. In all seven cases included in the material presented here, the dysfunction symptoms disappeared within 1 month.

The use of compression screws [45] is associated with greater impairment of facial nerve function, which results from the use of such fixations in difficult-to-treat fractures of the mandibular head [43] and some high-neck fractures. The weakening of the facial nerve is, of course, not a result of the use of these screws but the need to use them in a fracture requiring a wide opening of the region of the nerve branch course, a reduction that is a challenge in itself, along with the preservation of bone fragments during osteosynthesis [46]. A similar hypothesis was established by Marin et al., who noted that even when there are no visible signs of nerve injury, there is no guarantee that the nerve is actually intact. Due to friction and manipulation of the nerve branches during surgery, there is a possibility of nerve continuity damage, which can result in post-operative nerve dysfunction [47].

The authors believe that the type of fixing material used does not have a great impact on the development of surgical trauma but is indirectly related to mandibular anatomy and fracture pathology [48,49]. Simply put, plates and screws are selected according to the anatomical region that is damaged and not randomly. The results depend on the level of damage to the condyle and the difficulty of the surgery (as determined, for example, by the delay or duration of the procedure). The use of fixation material is secondary [22].

It is worth noting that the new computer-assisted design techniques have not become widespread in the discussed area of skeletal surgery. There are only two known applications of CAD/CAM in traumatology of the mandibular condylar process:

1. The Pavlychuk protocol developed for accurate reduction of mandibular head fractures [50,51];
2. Treatment of non-osteosynthesisable comminuted mandibular head fractures, i.e., design and manufacture of temporomandibular joint total alloplastic replacements [52,53,54].

So, the time when CAD/CAM techniques will support fracture treatment in this anatomically difficult region has not yet come.

The most clinically effective method of assessing the status of facial nerve disorders is the House–Brackmann scale. Moreover, it is crucial to determine the nature of facial nerve paralysis, specifically considering whether there is impairment of function without or with damage to the structure of the nerve branch. It is worth mentioning that, in the peripheral nervous system represented by the axolemma, nerves heal based on the breakdown and buildup of myelin and using the nutritional function of glial cells [55,56,57]. In accordance with nerve physiology, the initial 6 months of physiotherapy constitute the most efficacious post-operative treatment.

The initial element of improvement applied to patients is anti-oedema therapy (glucocorticosteroid treatment was administered only during the first few days after the operation) [58,59]. This treatment helps to improve circulation in the blood–lymphatic system [60,61]. Dexamethasone was given, not for its neuroprotective effect, but to reduce connective tissue oedema [62,63], especially in post-traumatic cases [64,65], and indeed, some believe that these drugs are harmful regardless of the extent of the injury [66,67], perhaps due to the inhibition of neuroprotective pathways [65].

Among the methods showing proven effectiveness are magnetotherapy treatments using low-frequency magnetic fields [68,69] and Kinesio Tape application [70,71,72]. Reconstruction proceeds based on the production of newly formed Schwann cells, in the myelin sheath of the peripheral fragment of the damaged nerve [73]. Moreover, research findings indicate that during the processes of nerve regeneration and reinnervation, a natural period of peak metabolic activity lasting approximately three weeks occurs. This period typically commences 3–4 weeks after the initial nerve injury [55,74]. The stimulation process should be continued until satisfactory functional results are obtained. It is recommended that a series of 10 to 20 treatments of a particular stimulus be administered, with a frequency of every day or every other day. The utilization of a maximum of two or three stimuli is recommended. The effects of the therapy are not typically noticed immediately; frequently, it requires approximately two to three weeks for the results of the stimulation to become visible. The optimal stimulation intervals with a specific stimulus, such as a magnetic field, should be 10 to 14 days between treatments. This interval may be utilised as a rest phase, in which no stimulation is employed, or alternatively, to avoid stagnation, local laser or electro stimulation treatments can be employed to stimulate the regenerative potential [56,64,75]. The stimulation of nerve regeneration is supported by the supplementation of vitamins, including B vitamins [76]. Vitamin B1 protects the energetic processes of the neuron and exhibits antioxidant abilities. Vitamin B6 can be used to support the synthesis of neurotransmitters. B12, on the other hand, is important in supporting the role of peripheral glial cells; it supports the survival of the neuron and is involved in the process of remyelination and nutrition of myelin cells [77].

The restoration of nerve cell function is typically observed within a period of 3 to 6 months under the conditions of an uninterrupted rehabilitation process. In the case of unfavourable circumstances, the time usually extends to a period of between 9 and 12 months or, in some cases, longer [78,79,80]. Nonetheless, if, following a period of 6–9 months, there has been no return of nerve function, the investigation focuses on determining whether there has been any interruption in the continuity of the nerve. When there are signs of improvement, the nerve should be still stimulated with no more than three stimuli. If there is no improvement, there is a need to consider reconstruction of the nerve with a cable graft or corrective plastic surgery before 24 months [81].

An interrupted nerve branch should be treated with surgical exploration and tension-free coaptation immediately. This gives predictable and good results, and steroid therapy can be used as a short-term, complementary method aimed at eliminating the swelling around the nerve [82].

It is also important to note that this scientific investigation included patients treated during the period of the COVID-19 pandemic. There was a specific time when rules in hospitals were changed and patients’ attitudes to treatment and following doctors’ recommendations were also modified due to many difficulties [83,84,85,86,87]. Many studies have revealed that during the pandemic, patients avoided medical appointments and sought medical help when they felt their health was in danger [87,88,89,90]. Therefore, the authors can hypothesise that patients without postsurgical complications avoided clinic appointments. These patients might have been excluded from this study due to a lack of medical history. Furthermore, during the pandemic, many appointments were changed to telemedicine consultations, during which patients had to perform self-examinations of their facial nerve function. In many cases, insufficient medical knowledge could result in misdiagnosis of the facial nerve function. The condition of the facial nerve during the first six months, when the most significant progress is observed, might easily be misdiagnosed. To sum up, the rate of facial nerve palsy might have been noted as higher than it actually was in the whole population. This problem can be considered a limitation of this study.

Finally, the authors would like to explain the high initial rate of observed dysfunction of the VII cranial nerve in the presented material (50.45%). First, in this study, there was a significant number of mandibular head and high-neck fractures (35% of all included cases) treated in an open manner. The surgeries performed for cases of fractures of the head and high-neck regions are surgical procedures on the TMJ. The rate of facial nerve palsy in this group is significantly higher than in cases of low-neck or base fracture treatment. Second, some authors may have missed a grade 2 paresis on the House–Brackmann scale (i.e., slight weakness, normal tone). The hypothesis is formed on the basis of the observation that a significant number of studies have failed to take into account the scale of the examination of the facial nerve function [43,91,92]. Third, there also seem to be significant methodological differences in the timing of the assessment of dysfunction, the criticality of the results obtained, and systematic long-term patient care (2–5 years). However, it remains promising to reduce residual dysfunction to 2 or 3 on the House–Brackmann scale and to a frequency of 1.82% of all patients treated in our study.

## 5. Conclusions

In conclusion, the results suggest that transient facial nerve palsy is a common complication (50.45%) of mandibular condylar fractures. Fortunately, in most cases (48.63%), it occurred as transient dysfunction; only 1.82% of palsies were permanent. Surgeons must be aware that the surgical approach can significantly impact post-operative facial nerve function. The safest approach is the retromandibular approach, which is recommended for use as often as possible and reasonable for fracture site visibility. The auricular and preauricular approaches are considered the most dangerous for the facial nerve and should be used with caution and only when necessary. Furthermore, physiotherapeutic treatment involving Kinesiotaping, magnetic fields, laser biostimulation, and vitamin B supplementation can resolve even serious post-operative facial nerve dysfunction.

It seems that the post-operational risk of permanent facial nerve palsy is relatively low. Thus, ORIF for mandibular condylar fracture can be offered to patients due to its many advantages, such as anatomical positioning of bone fragments, giving real hope of restoring occlusion, mouth opening and masticatory function.

## Figures and Tables

**Table 1:** The House–Brackmann scale, a nerve grading system developed in 1985 by Los Angeles otolaryngologist Dr. John W. House and Dr. Derald E. Brackmann.

Grade	Description	Eye Closure	Forehead Movement	Mouth Movement	Synkinesis
**1**	Normal facial function in all areas	Complete	Normal	Symmetrical	None
**2**	Slight weakness, normal tone, and symmetry at rest	Complete, slight effort	Normal	Slight asymmetry	Mild
**3**	Moderate dysfunction and no noticeable weakness at rest	Complete, with effort	Slightly reduced	Asymmetry present	Noticeable
**4**	Severe dysfunction and obvious facial weakness	Incomplete	No movement	Asymmetry with Effort	Present
**5**	Severe dysfunction and minimal facial motion	Incomplete	No movement	Very asymmetrical	Present
**6**	Total facial paralysis; no motion	Incomplete	No movement	No movement	None

**Table 2:** The original evaluation data for facial nerve dysfunction after open rigid internal fixation of mandibular condylar fracture. The numerical values correspond to the degrees of the House–Brachmann scale. Bold font number indicates the existence of statistically significant differences (*p* < 0.05) between variants of the variable. The values demonstrate mean ± SD (standard deviation).

Variable	Variants	00M	01M	02M	03M	04M	05M	06M	24M
Patients	Full Cohort	2.20 ± 1.25	1.98 ± 1.20	1.77 ± 1.07	1.54 ± 0.88	1.34 ± 0.69	1.19 ± 0.51	1.08 ± 0.37	1.02 ± 0.19
Sex	Female	**2.33 ± 1.20**	**2.24 ± 1.16**	**2.04 ± 1.09**	**1.65 ± 0.82**	1.40 ± 0.68	1.20 ± 0.48	1.08 ± 0.38	1.02 ± 0.16
	Male	**2.03 ± 1.27**	**1.90 ± 1.20**	**1.68 ± 1.05**	**1.51 ± 0.90**	1.32 ± 0.70	1.20 ± 0.52	1.09 ± 0.37	1.02 ± 0.20
Residence Place	Rural	2.26 ± 1.29	1.92 ± 1.19	1.69 ± 1.01	1.49 ± 0.85	1.27 ± 0.66	1.15 ± 0.44	1.05 ± 0.27	1.00 ± 0.00
	Urban	2.41 ± 1.32	2.01 ± 1.20	1.80 ± 1.09	1.56 ± 0.90	1.36 ± 0.71	1.21 ± 0.54	1.10 ± 0.41	1.03 ± 0.23
Injury Reason	Assault	**1.58 ± 0.95**	**1.51 ± 0.96**	**1.37 ± 0.84**	**1.23 ± 0.68**	**1.15 ± 0.53**	1.09 ± 0.40	1.06 ± 0.33	1.03 ± 0.26
	Fall	**2.48 ± 1.29**	**2.30 ± 1.22**	**2.06 ± 1.15**	**1.78 ± 0.97**	**1.50 ± 0.79**	1.27 ± 0.56	1.08 ± 0.31	1.02 ± 0.13
	Sports	1.33 ± 0.82	1.17 ± 0.41	1.17 ± 0.40	1.00 ± 0.00	1.00 ± 0.00	1.00 ± 0.00	1.00 ± 0.00	1.00 ± 0.00
	Vehicle	**2.30 ± 1.32**	**2.19 ± 1.24**	**1.89 ± 1.04**	**1.58 ± 0.81**	1.35 ± 0.65	1.20 ± 0.50	1.07 ± 0.38	1.00 ± 0.00
	Workplace	2.60 ± 1.51	2.60 ± 1.51	2.40 ± 1.35	2.20 ± 1.32	1.70 ± 0.95	1,50 ± 0.97	1.50 ± 0.97	1.20 ± 0.42
Intoxicants	No	2.20 ± 1.28	**2.09 ± 1.21**	1.83 ± 1.10	1.58 ± 0.88	1.34 ± 0.66	1.19 ± 0.51	1.06 ± 0.32	1.01 ± 0.10
	Yes	1.95 ± 1.21	**1.83 ± 1.16**	1.67 ± 1.03	1.49 ± 0.90	1.33 ± 0.74	1.20 ± 0.51	1.11 ± 0.44	1.04 ± 0.27
Fracture Diagnosis	CHF type A	2.50 ± 2.12	2.50 ± 2.12	2.00 ± 1.41	2.00 ± 1.41	2.00 ± 1.41	1.50 ± 0.71	1.00 ± 0.00	1.00 ± 0.00
	CHF type B	**3.29 ± 1.19**	**3.05 ± 1.12**	**2.57 ± 1.12**	**2.24 ± 1.09**	1.43 ± 0.81	1.29 ± 0.72	1.19 ± 0.68	1.00 ± 0.00
	CHF type C	**3.08 ± 1.09**	**2.92 ± 1.11**	**2.53 ± 1.15**	**2.19 ± 1.02**	**1.83 ± 0.93**	**1.49 ± 0.77**	1.23 ± 0.60	1.08 ± 0.35
	High-Neck	**3.15 ± 0.99**	**2.85 ± 1.07**	**2.54 ± 0.88**	1.85 ± 0.80	1.46 ± 0.66	1.38 ± 0.51	1.08 ± 0.29	1.00 ± 0.00
	Low-Neck	**2.19 ± 1.26**	**2.00 ± 1.08**	1.81 ± 1.09	**1.50 ± 0.84**	1.28 ± 0.58	1.15 ± 0.37	1.00 ± 0.00	1.00 ± 0.00
	Base	**1.46 ± 0.87**	**1.39 ± 0.84**	**1.30 ± 0.72**	**1.17 ± 0.52**	**1.11 ± 0.40**	**1.05 ± 0.24**	1.02 ± 0.15	1.01 ± 1.19
Condyle Fracture	Single	**1.88 ± 1.15**	**1.76 ± 1.06**	**1.55 ± 0.90**	**1.37 ± 0.72**	**1.20 ± 0.52**	**1.09 ± 0.30**	**1.02 ± 0.13**	**1.00 ± 0.00**
	Bilateral	**2.63 ± 1.34**	**2.52 ± 1.33**	**2.29 ± 1.24**	**1.95 ± 1.07**	**1.66 ± 0.92**	**1.43 ± 0.76**	**1.24 ± 0.63**	**1.08 ± 0.34**
Origin	Own Patient	**2.05 ± 1.24**	**1.93 ± 1.18**	**1.73 ± 1.06**	**1.51 ± 0.87**	1.32 ± 0.69	1.19 ± 0.51	1.09 ± 0.38	1.03 ± 1.20
	Another Centre	**2.70 ± 1.27**	**2.59 ± 1.22**	**2.26 ± 1.06**	**1.85 ± 0.95**	1.48 ± 0.70	1.26 ± 0.53	1.04 ± 0.20	1.00 ± 0.00
Surgical Approach	Extended Preauricular	**2.72 ± 1.18**	**2.56 ± 1.15**	**2.20 ± 1.09**	**1.78 ± 0.96**	**1.49 ± 0.75**	1.31 ± 0.62	1.16 ± 0.53	1.09 ± 0.36
	Preauricular	**2.52 ± 1.26**	**2.40 ± 1.22**	**2.09 ± 1.16**	**1.84 ± 1.02**	**1.53 ± 0.85**	1.27 ± 0.62	1.00 ± 0.00	1.00 ± 0.00
	Extended Retromadibular	**2.05 ± 1.34**	**1.93 ± 1.33**	1.78 ± 1.14	1.53 ± 0.82	1.33 ± 0.62	1.18 ± 0.38	1.03 ± 0.16	1.00 ± 0.00
	Auricular	**4.00 ± 0.00**	**3.67 ± 0.58**	**3.67 ± 0.58**	**3.33 ± 1.15**	3.00 ± 1.73	2.33 ± 1.15	2.00 ± 1.00	1.00 ± 0.00
	Retroauricular	2.63 ± 1.51	2.50 ± 1.41	2.25 ± 1.28	2.13 ± 1.13	1.50 ± 0.76	1.50 ± 0.76	1.13 ± 0.35	1.00 ± 0.00
	Retromandibular	**1.16 ± 0.46**	**1.11 ± 0.38**	**1.06 ± 0.28**	**1.01 ± 0.11**	**1.01 ± 0.11**	1.00 ± 0.00	1.00 ± 0.00	1.00 ± 0.00
	Periangular	1.38 ± 0.52	1.25 ± 0.46	1.13 ± 0.35	1.13 ± 0.35	1.00 ± 0.00	1.00 ± 0.00	1.00 ± 0.00	1.00 ± 0.00
	Intraoral	**1.53 ± 1.12**	**1.41 ± 0.80**	1.41 ± 0.80	1.18 ± 0.39	1.06 ± 0.24	1.00 ± 0.00	1.00 ± 0.00	1.00 ± 0.00
Comorbidity	5 Diseases	1.00 ± 0.00	1.00 ± 0.00	1.00 ± 0.00	1.00 ± 0.00	1.00 ± 0.00	1.00 ± 0.00	1.00 ± 0.00	1.00 ± 0.00
	4 Diseases	3.00 ± 0.00	3.00 ± 0.00	3.00 ± 0.00	2.00 ± 0.00	1.00 ± 0.00	1.00 ± 0.00	1.00 ± 0.00	1.00 ± 0.00
	3 Diseases	2.57 ± 1.51	2.43 ± 1.40	2.14 ± 1.07	1.86 ± 0.90	1.43 ± 0.53	1.29 ± 0.49	1.00 ± 0.00	1.00 ± 0.00
	2 Diseases	2.11 ± 1.32	2.00 ± 1.28	1.78 ± 1.17	1.56 ± 0.86	1.44 ± 0.78	1.17 ± 0.38	1.00 ± 0.00	1.00 ± 0.00
	1 Disease	2.45 ± 1.37	2.32 ± 1.32	2.05 ± 1.21	1.74 ± 1.02	1.53 ± 0.92	1.34 ± 0.70	1.16 ± 0.52	1.03 ± 0.16
	Healthy Generally	1.98 ± 1.19	1.86 ± 1.13	1.67 ± 1.00	1.47 ± 0.83	1.26 ± 0.58	1.14 ± 0.43	1.06 ± 0.32	1.03 ± 0.21
Fixing Material	Compressive Screws	**3.08 ± 1.07**	**2.89 ± 1.09**	**2.48 ± 1.10**	**2.13 ± 1.00**	**1.68 ± 0.86**	**1.40 ± 0.69**	1.18 ± 0.53	1.06 ± 0.31
	XCP	**1.25 ± 0.72**	**1.25 ± 0.72**	**1.20 ± 0.52**	**1.15 ± 0.37**	**1.00 ± 0.00**	1.00 ± 0.00	1.00 ± 0.00	1.00 ± 0.00
	ACP	**1.64 ± 1.02**	**1.55 ± 0.98**	**1.44 ± 0.92**	**1.26 ± 0.69**	**1.15 ± 0.52**	**1.07 ± 0.29**	1.02 ± 0.13	1.00 ± 0.00
	3 Straight Plates	2.20 ± 1.30	2.20 ± 1.30	1.60 ± 0.89	1.20 ± 0.45	1.20 ± 0.45	1.20 ± 0.45	1.00 ± 0.00	1.00 ± 0.00
	2 Straight Plates	**1.68 ± 1.31**	**1.57 ± 0.99**	**1.46 ± 0.88**	**1.30 ± 0.69**	**1.15 ± 0.60**	1.16 ± 0.50	1.09 ± 0.43	1.03 ± 0.17
	1 Straight Plate	2.00 ± 1.41	2.00 ± 1.41	1.50 ± 1.00	1.00 ± 0.00	1.00 ± 0.00	1.00 ± 0.00	1.00 ± 0.00	1.00 ± 0.00
Neurotmesis	Yes	**3.14 ± 1.07**	**3.00 ± 1.15**	2.29 ± 1.25	1.29 ± 1.25	1.86 ± 1.46	1.57 ± 1.13	1.43 ± 1.13	1.00 ± 0.00
	No	**2.08 ± 1.25**	**1.96 ± 1.19**	1.76 ± 1.07	1.52 ± 0.87	1.33 ± 0.67	1.19 ± 0.49	1.08 ± 0.34	1.03 ± 0.19
Mandible Fractures	4 Fractures	2.44 ± 1.74	2.44 ± 1.74	2.22 ± 1.48	1.89 ± 1.05	1.67 ± 0.87	1.33 ± 0.50	1.00 ± 0.00	1.00 ± 0.00
	3 Fractures	**2.36 ± 1.32**	**2.26 ± 1.27**	**2.11 ± 1.20**	**1.85 ± 1.06**	**1.58 ± 0.90**	1.36 ± 0.72	1.17 ± 0.54	1.05 ± 0.31
	2 Fractures	**1.70 ± 1.10**	**1.60 ± 1.07**	**1.45 ± 0.90**	**1.29 ± 0.69**	**1.19 ± 0.52**	1.11 ± 0.40	1.07 ± 0.35	1.03 ± 0.17
	Only Condyle	**2.39 ± 1.22**	**2.22 ± 1.20**	**1.77 ± 1.02**	1.58 ± 0.84	1.31 ± 0.61	1.15 ± 0.38	1.08 ± 0.37	1.00 ± 0.00

Abbreviations: 00M—immediately post-op; 01M, 02M, 03M, 04M, 05M, 06M, and 24M—1, 2, 3, 4, 5, 6, and 24 months post-op; CHF—Condylar Head fracture.

**Table 3:** Number of patients affected by facial nerve dysfunction in each type of condylar fracture.

Type of Condylar Fracture	No. of Patients with Transient Facial Nerve Palsy	Transient Facial Nerve Palsy (%)	No. of Patients with Permanent Facial Nerve Palsy	Total Sample Size
Head	83	82%	4 *	101
High-neck	12	92%	0	13
Low-neck	18	56%	0	32
Base	47	26%	2 *	183
**All**	**160**	**48.63%**	**6**	**329**

* The observed patients suffered grade 2 or 3 based on the House–Brackmann scale.

**Table 4:** Number of patients affected by facial nerve dysfunction in each type of surgical approach used.

Approach	No. of Patients with Transient Facial Nerve Palsy	Transient Facial Nerve Palsy (%)	No. of Patients with Permanent Facial Nerve Palsy	Total Sample Size
Preauricular	56	69%	0	81
Extended Preauricular	66	69%	6 *	87
Auricular	3	100%	0	3
Retroauricular	5	63%	0	8
Retromandibular	11	13%	0	85
Extended Retromandibular	18	45%	0	40
Periangular	3	38%	0	8
Intraoral	4	24%	0	17
**All**	**166**	**49%**	**6**	**329**

* The observed patients suffered grade 2 or 3 based on the House–Brackmann scale.

## Data Availability

The data on which this study is based will be made available upon request at https://www.researchgate.net/profile/Marcin-Kozakiewicz (accessed on 21 July 2025) and www.youtube.com/@marcinkozakiewicz5618 (access on 21 July 2025).

## Abbreviations

The following abbreviations are used in this manuscript:

TMJ	Temporomandibular joint
CHF	Condylar head fracture
ORIF	Open rigid internal fixation
ACP	“A”-shape condylar plate
XCP	“X”-shape condylar plate
M	Month
CC	Correlation coefficient
IMF	Intramaxillary fixation

## References

[1] Kolk A., Scheunemann L.M., Grill F., Stimmer H., Wolff K.D., Neff A. (2020). Prognostic Factors for Long-Term Results after Condylar Head Fractures: A Comparative Study of Non-Surgical Treatment versus Open Reduction and Osteosynthesis. J. Cranio-Maxillofac. Surg..

[2] Bhutia O., Kumar L., Jose A., Roychoudhury A., Trikha A. (2014). Evaluation of Facial Nerve Following Open Reduction and Internal Fixation of Subcondylar Fracture through Retromandibular Transparotid Approach. Br. J. Oral Maxillofac. Surg..

[3] Ellis E., McFadden D., Simon P., Throckmorton G. (2000). Surgical Complications with Open Treatment of Mandibular Condylar Process Fractures. J. Oral Maxillofac. Surg..

[4] Gibson A.C., Merrill T.B., Boyette J.R. (2023). Complications of Mandibular Fracture Repair. Otolaryngol. Clin. N. Am..

[5] Korzon T. (1966). The Issue of the Advisability of Surgical Treatment of Mandibular Condylar Process Fractures in the Light of Clinical and Experimental Studies. Postdoctoral Thesis.

[6] Minervini G., Franco R., Marrapodi M.M., Di Blasio M., Isola G., Cicciù M. (2023). Conservative Treatment of Temporomandibular Joint Condylar Fractures: A Systematic Review Conducted According to PRISMA Guidelines and the Cochrane Handbook for Systematic Reviews of Interventions. J. Oral Rehabil..

[7] Monarchi G., Catarzi L., Paglianiti M., Valassina D., Balercia P., Consorti G. (2024). A Comparative Analysis of Surgical and Conservative Management in Intra-Articular Condylar Fractures: A Retrospective Study. Surgeries.

[8] Zide M.F., Kent J.N. (1983). Indications for open reduction of mandibular condyle fractures. J. Oral Maxillofac. Surg..

[9] Kozakiewicz M. (2019). Fractures of Mandible Condyle Process.

[10] Koch S. (2003). Biomechanische Untersuchungen zur Stabilität Verschiedener Osteosynthesematerialien bei Gelenkwalzenfrakturen vom Typ A, Klinik und Poliklinik für Mund-Kiefer-Gesichtschirurgie der Technischen Universität München Klinikum Rechts der Isar. Ph.D. Thesis.

[11] Kolk A., Neff A. (2015). Long-Term Results of ORIF of Condylar Head Fractures of the Mandible: A Prospective 5-Year Follow-Up Study of Small-Fragment Positional-Screw Osteosynthesis (SFPSO). J. Craniomaxillofac. Surg..

[12] Kozakiewicz M., Okulski J., Krasowski M., Konieczny B., Zieliński R. (2023). Which of 51 Plate Designs Can Most Stably Fixate the Fragments in a Fracture of the Mandibular Condyle Base?. J. Clin. Med..

[13] Kozakiewicz M., Sołtysiak P. (2017). Pullout force comparison of selected screws for rigid fixation in maxillofacial surgery. Dent. Med. Probl..

[14] Kozakiewicz M. (2019). Comparison of compression screws used for mandible head fracture treatment—Experimental study. Clin. Oral Investig..

[15] Kozakiewicz M. (2018). Small-diameter compression screws completely embedded in bone for rigid internal fixation of the condylar head of the mandible. Br. J. Oral Maxillofac. Surg..

[16] Kozakiewicz M., Gabryelczak I. (2022). Bone Union Quality after Fracture Fixation of Mandibular Head with Compression Magnesium Screws. Materials.

[17] Neff A., Neff F., Kolk A., Horch H.H. (2001). Risiken und perioperative Komplikationen bei offenen gelenkchirurgischen Eingriffen. Dtsch. Zahnärztl. Z..

[18] Ghezta N.K., Ram R., Bhardwaj Y., Sreevidya S., Sharma M., Bhatt R. (2021). Operator Experience and Fracture Location Affects the Rate of Facial Nerve Injury in Condylar Fractures: An Analysis of 89 Cases. J. Oral Maxillofac. Surg..

[19] Sapna T., Vishal V., Mohd R., Saurabh S., Kumar S.A., Kumar S.N. (2022). Is the facial nerve at risk following surgical correction of mandibular condylar fracture: A systematic review and meta-analysis. Nat. J. Maxillofac. Surg..

[20] Elmadawy A., Hegab A., Alahmady H., Shuman M. (2015). Clinical and electromyographic assessment of facial nerve function after temporomandibular joint surgery. Int. J. Oral Maxillofac. Surg..

[21] Rajasekhar G., Kruthi N., Nandagopl V., Sudhir R. (2014). Retrospective Study of Facial Nerve Injury in Temporomandibular Joint Surgeries Following Preauricular Approach. Surg. Curr. Res..

[22] Al-Moraissi E.A., Ellis E., Neff A. (2018). Does Encountering the Facial Nerve during Surgical Management of Mandibular Condylar Process Fractures Increase the Risk of Facial Nerve Weakness? A Systematic Review and Meta-Regression Analysis. J. Craniomaxillofac. Surg..

[23] Moin A., Shetty A.D., Archana T.S., Kale S.G. (2018). Facial nerve injury in temporomandibular joint approaches. Ann. Maxillofac. Surg..

[24] Hunt B.R., Johnson S.A., Klukkert Z.S. (2025). Distribution, scaling, and depiction of the temporal branches of the facial nerve. Sci. Rep..

[25] Chan J.Y., Byrne P.J. (2011). Management of facial paralysis in the 21st century. Facial Plast. Surg..

[26] Tollefson T.T., Hadlock T.A., Lighthall J.G. (2018). Facial Paralysis Discussion and Debate. Facial Plast. Surg..

[27] Noguera C.M., Noguera M.D. (2023). Management of Facial Nerve Palsy: Part Two of a Two-Part Series Exploring the Diagnosis and Management of Facial Paralysis. Rev. Ophthalmol..

[28] von Elm E., Altman D.G., Egger M., Pocock S.J., Gotzsche P.C., Vandenbrouche J.P., STROBE Initiative (2007). The Strengthening the Reporting of Observational Studies in Epidemiology (STROBE) Statement: Guidelines for Reporting Observational Studies. Lancet.

[29] Kozakiewicz M. (2019). Classification proposal for fractures of the processus condylaris mandibulae. Clin. Oral Investig..

[30] Neff A., Cornelius C.P., Rasse M., Torre D.D., Audigé L. (2014). The Comprehensive AOCMF Classification System: Condylar Process Fractures—Level 3 Tutorial. Craniomaxillofac. Trauma Reconstr..

[31] Loukota R.A., Rasse M. (2010). Nomenclature/classification of fractures of the mandibular condylar head. Br. J. Oral Maxillofac. Surg..

[32] Yang H.-M., Yoo Y.-B. (2014). Anatomy of the Facial Nerve at the Condylar Area: Measurement Study and Clinical Implications. Sci. World J..

[33] Imai T., Fujita Y., Takaoka H., Motoki A., Kanesaki T., Ota Y., Chisoku H., Ohmae M., Sumi T., Nakazawa M. (2020). Longitudinal Study of Risk for Facial Nerve Injury in Mandibular Condyle Fracture Surgery: Marginal Mandibular Branch-Traversing Classification of Percutaneous Approaches. Clin. Oral Investig..

[34] Kozakiewicz M., Walczyk A. (2023). Current Frequency of Mandibular Condylar Process Fractures. J. Clin. Med..

[35] Kozakiewicz M., Zieliński R., Krasowski M., Okulski J. (2019). Forces Causing One-Millimeter Displacement of Bone Fragments of Condylar Base Fractures of the Mandible after Fixation by All Available Plate Designs. Materials.

[36] Sikora M., Chęciński M., Nowak Z., Chęcińska K., Olszowski T., Chlubek D. (2021). The Use of Titanium 3D Mini-Plates in the Surgical Treatment of Fractures of the Mandibular Condyle: A Systematic Review and Meta-Analysis of Clinical Trials. J. Clin. Med..

[37] Kermer C., Undt G., Rasse M. (1998). Surgical reduction and fixation of intracapsular condylar fractures; A follow-up study. Int. J. Oral Maxillofac. Surg..

[38] Neff A., Kolk A., Meschke F., Deppe H., Horch H.H. (2005). Kleinfragmentschrauben vs. Plattenosteosynthese bei Gelenkwalzenfrakturen; Vergleich funktioneller Ergebnisse mit MRT und Achsiographie Small fragment screws vs. plate osteosynthesis in condylar head fractures. Mund Kiefer Gesichtschir..

[39] Kozakiewicz M., Świniarski J. (2017). Finite element analysis of newly introduced plates for mandibular condyle neck fracture treatment by open reduction and rigid fixation. Dent. Med. Probl..

[40] Rahnama M. (2024). Oral and Maxillofacial Surgery.

[41] Pulino B., de Andrade J.F., Felippe T., Prestes F., Neff A., Guerra R.C. (2025). Surgical approaches for condylar fractures: An analysis of the advantages of transmeatal retroauricular access. Adv. Oral Maxillofac. Surg..

[42] Kanno T. (2020). Surgical Approaches to Open Reduction and Internal Fixation of Mandibular Condylar Fractures. Shimane J. Med. Sci..

[43] Becker P., Bouffleur F., Heimes D., Theim D.G.E., Seifert L.B., Neff A., Wiltfang J., Heiland M., Kesting M., Bar A.-K. (2025). Facial trauma management: A nationwide data collection on practice patterns and patient care in oral and maxillofacial surgery in Germany. J. Craniomaxillofac. Surg..

[44] García-Guerrero I., Ramírez J.M., de Diego R.G., Martínez-González J.M., Poblador M.S., Lancho J.L. (2018). Complications in the treatment of mandibular condylar fractures: Surgical versus conservative treatment. Ann. Anat..

[45] Kozakiewicz M., Gabryelczak I. (2022). The Osteosynthesis of the Mandibular Head, Does the Way the Screws Are Positioned Matter?. J. Clin. Med..

[46] Antoniadis G., Kretschmer T., Pedro M.T., König R.W., Heinen C.P., Richter H.P. (2014). Iatrogenic nerve injuries: Prevalence, diagnosis and treatment. Dtsch. Arztebl. Int..

[47] Marin P., Pouliot D., Fradet G. (2011). Facial nerve outcome with a preoperative stimulation threshold under 0.05 mA. Laryngoscope.

[48] Neff A. (2019). Open reduction and internal fixation in temporomandibular joint traumatology: Current concepts and future perspectives. Stomatol. Dis. Sci..

[49] Al-Moraissi E.A., Neff A., Kaur A., Falci S.G.M., de Souza G.M., Ellis E. (2023). Treatment for Adult Mandibular Condylar Process Fractures: A Network Meta-Analysis of Randomized Clinical Trials. J. Oral Maxillofac. Surg..

[50] Pavlychuk T., Chernogorskyi D., Chepurnyi Y., Neff A., Kopchak A. (2020). Application of CAD/CAM Technology for Surgical Treatment of Condylar Head Fractures: A Preliminary Study. J. Oral Biol. Craniofac. Res..

[51] Pavlychuk T., Chernogorskyi D., Chepurnyi Y., Neff A., Kopchak A. (2020). Biomechanical Evaluation of Type P Condylar Head Osteosynthesis Using Conventional Small-Fragment Screws Reinforced by a Patient-Specific Two-Component Plate. Head Face Med..

[52] Pruszyńska P., Kozakiewicz M., Szymor P., Wach T. (2024). Personalized Temporomandibular Joint Total Alloplastic Replacement as a Solution to Help Patients with Non-Osteosynthesizable Comminuted Mandibular Head Fractures. J. Clin. Med..

[53] Da Rosa E.L., Oleskovicz C.F., Aragão B.N. (2004). Rapid Prototyping in Maxillofacial Surgery and Traumatology: Case Report. Braz. Dent. J..

[54] Sinno H., Tahiri Y., Gilardino M., Bobyn D. (2010). Engineering Alloplastic Temporomandibular Joint Replacements. Mcgill J. Med..

[55] Hirose T., Shiozaki T., Shimizu K., Tamoyoshi M., Noguchi K., Ohnishi M., Shimazu T. (2013). The Effect of Electrical Muscle Stimulation on the Prevention of Disuse Muscle Atrophy in Patients with Consciousness Disturbance in the Intensive Care Unit. J. Crit. Care.

[56] Miller R.C., Brumback R.A., Gerst J. (1984). The effects of nerve injury on the neuromuscular junction. The Neuromuscular Junction.

[57] Rola R. (2012). Physiological basis of electrophysiological studies of the peripheral nervous system. Postgrad. Neurol..

[58] Bravo M., Kohan J.B., Monasterio M.U. (2022). Effectiveness of Glucocorticoids in Orthognathic Surgery: An Overview of Systematic Reviews. Br. J. Oral Maxillofac. Surg..

[59] Oksa M., Haapanen A., Furuholm J., Thorén H., Snäll J. (2021). Effect of Perioperative Systemic Dexamethasone on Pain, Edema, and Trismus in Mandibular Fracture Surgery: A Randomized Trial. J. Craniofac. Surg..

[60] Okano M. (2009). Mechanisms and clinical implications of glucocorticosteroids in the treatment of allergic rhinitis. Clin. Exp. Immunol..

[61] Seo K.H. (2021). Perioperative glucocorticoid management based on current evidence. Anesth. Pain Med..

[62] Semper-Hogg W., Fuessinger M.A., Dirlewanger T.W., Cornelius C.P., Metzger M.C. (2017). The influence of dexamethasone on postoperative swelling and neurosensory disturbances after orthognathic surgery: A randomized controlled clinical trial. Head Face Med..

[63] Scheller K., Scheller C. (2014). Nimodipine for peripheral nerve recovery after maxillofacial and vestibular schwannoma surgery. Muscle Nerve.

[64] Robertson V., Ward A., Low J., Reed A. (2009). Physical Therapy: Clinical and Biophysical Aspects.

[65] Levin L.A., Beck R.W., Joseph M.P., Seiff S., Kraker R. (1999). The treatment of traumatic optic neuropathy: The International Optic Nerve Trauma Study. Ophthalmology.

[66] Edwards P., Arango M., Balica L., Cottingham R., El-Sayed H., Farrell B., Fernandes J., Gogichaisvili T., Golden N., Hartzenberg B. (2005). Final results of MRC CRASH, a randomised placebo-controlled trial of intravenous corticosteroid in adults with head injury—Outcomes at 6 months. Lancet.

[67] Zhu N., Xiang B., Shi J., Yang P., Dai Y., Wang S. (2024). The Effect of Perineural Dexamethasone on Nerve Injury and Recovery of Nerve Function after Surgery: A Randomized Controlled Trial. Heliyon.

[68] Widłak P., Tomczyk Ł., Woldańska-Okońska M., Bartnicki P. (2024). Effect of low-frequency magnetic field (magnetic stimulation) and kinesitherapy on the level of selected blood parameters in haemodialysis patients. Med. Res. J..

[69] Friscia M., Abbate V., De Fazio G.R., Montemurro N., Guglielmi V., Marenzi G., Tartaglia G.M. (2024). Pulsed Electromagnetic Fields (PEMF) as a Valid Tool in Orthognathic Surgery to Reduce Post-Operative Pain and Swelling: A Prospective Study. Oral Maxillofac. Surg..

[70] Sobiech M., Czępińska A., Zieliński G., Zawadka M., Gawda P. (2022). Does Application of Lymphatic Drainage with Kinesiology Taping Have Any Effect on the Extent of Edema and Range of Motion in Early Postoperative Recovery following Primary Endoprosthetics of the Knee Joint?. J. Clin. Med..

[71] Tozzi U., Santagata M., Sellitto A., Tartaro G.P. (2016). Influence of Kinesiologic Tape on Post-Operative Swelling after Orthognathic Surgery. J. Maxillofac. Oral Surg..

[72] Lietz-Kijak D., Kijak E., Krajczy M., Bogacz K., Łuniewski J., Szczegielniak J. (2018). The Impact of the Use of Kinesio Taping Method on the Reduction of Swelling in Patients after Orthognathic Surgery: A Pilot Study. Med. Sci. Monit..

[73] Skowroński J. (2025). Surgery of the Peripheral Nerves.

[74] Kandel E.R., Schwartz J.H., Jessell T.M. (2000). Principles of Neural Science.

[75] Łukasik A. (2007). Fundamentals of electro diagnosis in peripheral nerve palsy. Balneology.

[76] Pinzon R.T., Schellack N., Matawaran B.J., Tsang M.W., Deerochanawong C., Hiew F.L., Nafach J., Khadilkar S. (2023). Clinical Recommendations for the Use of Neurotropic B Vitamins (B1, B6, and B12) for the Management of Peripheral Neuropathy: Consensus from a Multidisciplinary Expert Panel. J. Assoc. Physicians India.

[77] Calderón-Ospina C.A., Nava-Mesa M.O. (2020). B Vitamins in the Nervous System: Current Knowledge of the Biochemical Modes of Action and Synergies of Thiamine, Pyridoxine, and Cobalamin. CNS Neurosci. Ther..

[78] Błaszczyk J.W. (2004). Clinical Biomechanics.

[79] Traczyk W.Z. (2005). Human Physiology in Outline.

[80] Shandrin I.Y., Khodabukus A., Bursac N. (2016). Striated Muscle Function, Regeneration, and Repair. Cell. Mol. Life Sci..

[81] Mehta M. (2023). Atlas of Facial Nerve Surgery and Reanimation Procedures.

[82] Greiner R.C., Kohlberg G.D., Lu G.N. (2024). Management of Facial Nerve Trauma. Curr. Opin. Otolaryngol. Head Neck Surg..

[83] Davis T.S., Lou F., Xie S.J., Mouro-Fuentes E.A., Rodrigues E.B. (2023). Evaluating Adherence to Diabetic Retinopathy Care in an Urban Ophthalmology Clinic Utilizing the Compliance With Annual Diabetic Eye Exams Survey. Cureus.

[84] Pogany L., Horvath A.A., Slezak A., Rozsavolgyi E., Lazary J. (2020). A COVID-19 járvány miatt elrendelt első veszélyhelyzet a pszichiátriai betegek szemszögéből: Gondozói felmérés; The first lockdown due to COVID-19 pandemic from the psychiatric patients’ perspective: An ambulatory care client experience survey. Neuropsychopharmacol. Hung..

[85] Iorga M., Soponaru C., Socolov R.V., Cărăuleanu A., Socolov D.G. (2021). How the SARS-CoV-2 Pandemic Period Influenced the Health Status and Determined Changes in Professional Practice among Obstetrics and Gynecology Doctors in Romania. Medicina.

[86] Cha H.J., Jeon M.K. (2024). Experience of Family Caregivers in Long-Term Care Hospitals During the Early Stages of COVID-19: A Phenomenological Analysis. Healthcare.

[87] Bielecki-Kowalski B., Kowalczyk O., Podziewska M., Agier P., Kroc-Szczepkowska A., Kozakiewicz M. (2024). The Evaluation of Oral Health in Patients Undergoing Dental Treatment During the COVID-19 Pandemic. J. Clin. Med..

[88] McMaster T., Wright T., Mori K., Stelmach W., To H. (2021). Current and future use of telemedicine in surgical clinics during and beyond COVID-19: A narrative review. Ann. Med. Surg..

[89] Puyo E.M., Salvati L.R., Garg N., Baylay H., Kariveda R.R., Carnino J.M., Nathan A.J., Levi J.R. (2025). The impact of COVID-19 and socioeconomic determinants on appointment non-attendance in an urban otolaryngology clinic: A retrospective analysis from a safety net hospital. Ann. Otol. Rhinol. Laryngol..

[90] Mason M.C., Vedhanayagam K., Jernigan J.A. (2021). Evaluating patient adherence to routine and symptom indicated colonoscopies during the COVID-19 pandemic. Cureus.

[91] Handschel J., Rüggeberg T., Depprich R., Schwartz F., Meyer U., Kubler N.R., Naujkos C. (2012). Comparison of various approaches for the treatment of fractures of the mandibular condylar process. J. Craniomaxillofac. Surg..

[92] Franke A., Matschke J.B., Sembdner P., Seidler A., McLeod N.M.H., Leonhardt H. (2025). Long-term outcomes of open treatment of condylar head fractures using cannulated headless bone screws—A retrospective analysis. Int. J. Oral Maxillofac. Surg..

